# Gene Expression Profiling of the Optic Nerve Head of Patients with Primary Open-Angle Glaucoma

**DOI:** 10.1155/2017/6896390

**Published:** 2017-04-05

**Authors:** Xinrong Wang, Ke Gong, Haiyan Li, Congyi Wang, Chaoyi Qu, Hui Li

**Affiliations:** ^1^Department of Ophthalmology, The Fourth Hospital of Xi'an, Xi'an, Shaanxi 710004, China; ^2^Department of Endocrinology, Shaanxi Provincial People's Hospital, Xi'an, Shaanxi 710068, China

## Abstract

*Background*. The pressure-induced axonal injury of the vulnerable ONH has led many researchers to view glaucoma from the perspective of the genetic basis of the angle of the ONH. However, genetic studies on POAG from this perspective are limited. *Methods*. Microarray dataset GSE45570 of the ONH of healthy individuals and POAG patients were downloaded from the Gene Expression Omnibus. After screening for the DEGs using the limma package, enrichment analysis was performed using DAVID. The DEG interaction network was constructed using cancer spider at BioProfiling.de. Thereafter, DEG-related TFs were predicted using TRANSFAC, and TF-DEG regulatory networks were visualized using Cytoscape. *Results*. Thirty-one DEGs were identified including 11 upregulated and 20 downregulated DEGs. Thereafter, gene ontology terms of nucleosome assembly, sensory perception and cognition, and pathway of signaling by GPCR were found to be enriched among the DEGs. Furthermore, DEG interaction and TF-DEG networks were constructed. NEUROD1 was present in both the DEG network and the TF-DEG network as the node with the highest degree and was predicted as a marker gene in the ONH of patients with POAG. *Conclusion*. NEUROD1 may contribute greatly to the ONH of patients with POAG and was found to be involved in eye development and diseases.

## 1. Background

After cataracts, glaucoma is the second-leading cause of blindness worldwide [1], and it has been classified into specific types, including primary glaucoma and its variants [2]. Primary open-angle glaucoma (POAG) is defined as a progressive optic neuropathy with acquired loss of optic nerve fibers [3]. The characteristics of insidious onset and irreversible blindness caused by POAG have made it a major worldwide health concern [4]. As the most common type of glaucoma, POAG accounts for approximately 60–70% cases of glaucoma [5]. Therefore, understanding the mechanisms of POAG and developing novel therapeutic strategies are of great importance.

The altering evacuation of the aqueous humor in POAG always causes an increase in intraocular pressure and causes anatomical damage to the optic nerve. The optic nerve head (ONH) is the site where the optic nerve forms a ganglion with cell axons [6]. It is the likely site of initial damage, including axonal cytoskeleton damage, axonal transport disruption, and putative axonal regeneration, in the glaucomatous eye [7]. Furthermore, the fortified astrocytes of ONH are the targets of elevated intraocular pressure [8]. The pressure-induced axonal injury of the vulnerable ONH has led many researchers to view glaucoma from the perspective of the genetic basis of the ONH. Optic nerve degeneration in glaucoma was observed in individuals harboring common variants of the 9p21 and 8q22 loci [9]. Moreover, interleukin-6-type cytokine signaling are associated with gene expression responses in early ONH injury in rat model of glaucoma [10]. Transforming growth factor-*β*2 is also involved in glaucomatous damage to the ONH [11]. However, genetic studies of POAG from the perspective of ONH are limited.

In this study, differentially expressed genes (DEGs) in normal and POAG ONH samples were identified. Thereafter, enrichment analysis, DEG interaction network construction, transcription factor (TF) prediction, and TF-DEG network analyses were performed successively. These bioinformatics analyses may be useful in identifying ONH marker genes in patients with POAG and provide a basis for developing new therapies for this disease.

## 2. Materials and Methods

### 2.1. Microarray Data

Gene expression profiling data of GSE45570 was downloaded from the Gene Expression Omnibus (GEO) database (http://www.ncbi.nlm.nih.gov/geo/) based on the GPL5175 Affymetrix human exon 1.0 ST array. Six normal ONH samples (mean age = 87.67) and 6 ONH samples from patients with POAG (mean age = 88.83) were collected.

### 2.2. Data Preprocessing and Screening for DEGs

The probes in gene expression matrix were converted into gene names according to the platform annotation information. The expression values of the probes mapped to a given gene were averaged and considered as the final gene expression value for each sample. Thereafter, the missing data were imputed by the K-nearest neighbor (KNN) method using the impute package of R [12], and median normalization was performed using the preprocessCore package of R [13]. Finally, the limma package in R [13] was used to identify significant DEGs in the POAG ONH and normal ONH samples. *P* value < 0.05 and |log2 fold change (FC)| > 0.585 were required for each DEGs.

### 2.3. Pathway and Functional Enrichment Analyses

The Database for Annotation, Visualization, and Integrated Discovery (DAVID) provides a comprehensive set of functional annotation tools for investigators to understand the biological relevance of genes [14]. In this study, DAVID was applied to investigate the main Gene Ontology (GO) functions and the Kyoto Encyclopedia of Genes and Genomes (KEGG) pathways of DEGs involved in POAG. *P* value < 0.05 was chosen as the cut-off criterion.

### 2.4. DEG Interaction Network Analysis

BioProfiling.de (http://www.bioprofiling.de/CCancer.html) provides a common interface for a collection of current analytical tools used in genomics, proteomics, and metabolomics studies. In this study, CCancer spider in BioProfiling.de was applied to implement a global network statistical framework for analyzing mined DEGs and their correlative genes in ONH samples of patients with POAG. The DEG interaction network was obtained with the cut-off criteria of *P* value < 0.01 based on the hypergeometric distribution test.

### 2.5. TF-DEG Network Construction

The DEGs in the ONH of POAG samples were aligned and annotated with the gene symbols in the TRANSFAC database [15] to screen for potential TFs. DEG-related binding sites for TFs were predicted using the University of California Santa Cruz (UCSC) database. Finally, the TF-DEG network was visualized using Cytoscape [16].

## 3. Results

### 3.1. Data Preprocessing and DEG Screening

**Figure 1:**
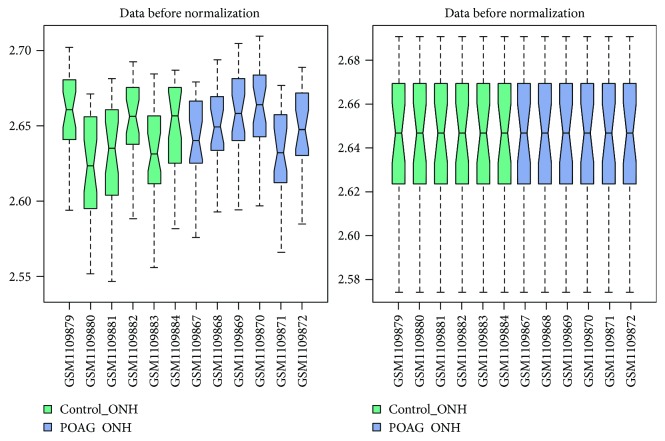
Microarray chips normalization.

The normalized microarray data is shown in Figure 1. All boxes representing gene expression values of samples were centered on a straight line after median normalization of microarray data. Thirty-one significant DEGs were identified after screening of DEGs using *P* value < 0.05 and |log2 FC| > 0.585 as the cut-off criteria (Table 1). Among them, there were 11 upregulated DEGs such as the prostaglandin-endoperoxide synthase 2 (*PTGS2*) gene. Meanwhile, 20 other genes were downregulated, such as the eyes shut homolog (*EYS*) and interphotoreceptor matrix proteoglycan 2 (*IMPG2*) genes.

### 3.2. Functional and Pathway Enrichment Analyses

GO function (*P* value < 0.05) and KEGG pathway (*P* value < 0.05) enrichment analyses were performed to investigate the specific functions associated with DEGs. As shown in Table 2, the upregulated genes were mainly enriched in the terms of nucleosome assembly and chromatin assembly, whereas genes involved in sensory perception and cognition were mainly downregulated. Moreover, signaling by GPCR and olfactory transduction pathways were enriched in the downregulated DEGs. It is noteworthy that the guanine nucleotide-binding protein (G protein) alpha-transducing activity polypeptide 2 (*GNAT2*) and retinol-binding protein 3 (*RBP3*) were jointly and significantly enriched in GO terms of sensory perception, cognition, and signaling by GPCR pathway.

### 3.3. DEG Interaction Network

**Figure 2:**
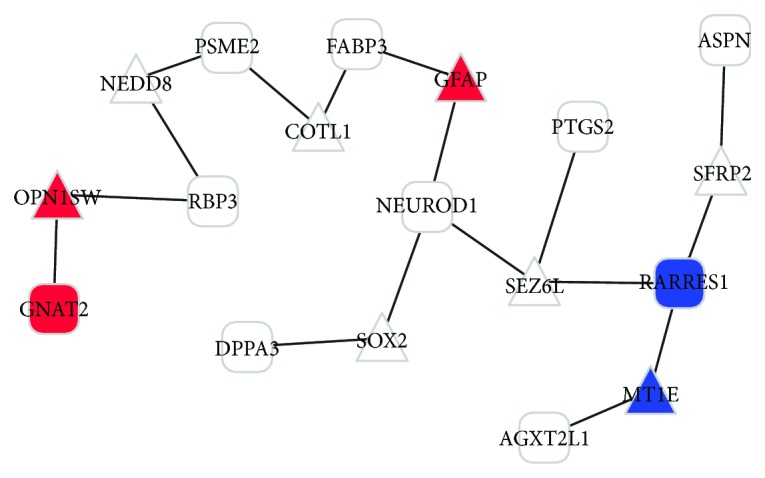
Differentially expressed genes (DEG) interaction network. Squares represent inputted DEGs; triangles represent intermediate genes recorded in BioProfiling.de; red nodes represent disease-related genes; blue nodes represent carcinoma-related genes.

The associations of DEGs and their related genes were analyzed by CCancer spider at BioProfiling.de. Ten DEGs and 8 other closely related genes were identified and the DEG interaction network was obtained (*P* value < 0.01) (Figure 2). In this network, the downregulated *GNAT2* was a disease-related gene, and it was linked with *RBP3* by the short-wave-sensitive opsin 1 (*OPN1SW*).

### 3.4. TF-DEG Network

**Figure 3:**
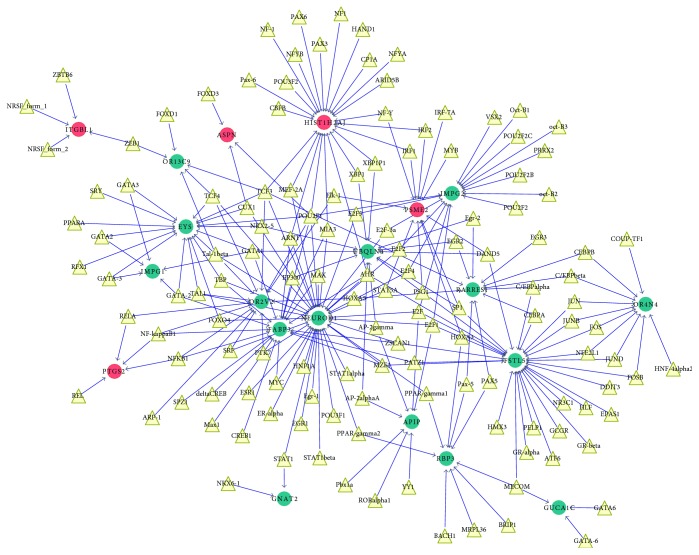
Transcription factor (TF) differentially expressed genes (DEG) network. Red nodes represent upregulated DEGs; green nodes represent downregulated DEGs; yellow triangles represent TFs; arrowed lines represent the regulatory relationship.

TFs of the DEGs were predicted based on TRANSFAC and UCSC. The TF-DEG network was constructed upon integrating DEGs and TFs (Figure 3). The neuronal differentiation 1 (*NEUROD1*) with the highest node degree of 36 was located in the center of the network.

## 4. Discussion

Gene expression profiling was systematically analyzed in this study to gain insight into the molecular mechanism of POAG from the perspective of the genetic basis of the ONH. Consequently, 31 DEGs were screened, including 11 upregulated and 20 downregulated genes. Based on pathway and functional enrichment analyses, genes involved in nucleosome assembly, sensory perception, and cognition were enriched by these DEGs. Further, the DEG interaction network and the TF-DEG network analyses indicated that *NEUROD1* might be a marker gene in the ONH of patients with POAG.

Certain DEGs, such as *EYS*, *PTGS2*, and *IMPG2*, identified in this study have been demonstrated to be associated with ophthalmic diseases and might participate in the development of POAG. The downregulated *EYS* was enriched in functional terms of visual perception and sensory perception of light stimulus. *EYS* is expressed in the photoreceptor layer of the retina, and a mutation in *EYS* plays a role in autosomal recessive retinitis pigmentosa [17]. Moreover, upregulated *PTGS2* found in our study encodes a cyclooxygenase, which acts both as a peroxidase and as a dioxygenase [18]. It was reported that *PTGS2*, located on chromosome 1q23–q25, might be related to POAG (GLC1A) [19]. Meanwhile, *IMPG2*, encoding a proteoglycan, contributes to the development of the interphotoreceptor matrix and may play a role in the maintenance and growth of the light-sensitive photoreceptor [20]. Diseases including maculopathy and retinitis pigmentosa type 56 are associated with this gene [21]. All these findings suggest that the DEGs identified in the ONH might be involved in the development of POAG.

The DEG interaction network demonstrated that genes such as *GNAT2* and *RBP3* which were linked with *OPN1SW* may be potential markers and may be jointly regulated genes in the ONH of patients with POAG. These genes were jointly enriched in the significant GO functional terms including sensory perception and cognition. Remarkably, *GNAT2* is a disease-related gene, as identified in this study. During visual impulses, the coupling of cGMP-phosphodiesterase and rhodopsin transducin is activated by transducin [22]. The alpha subunit of transducin is encoded by *GNAT2* [23], and evidence shows that *GNAT2* is involved in diseases such as achromatopsia [24] and oligocone trichromacy [25]. Moreover, *GNAT2* and *OPN1SW* are cone-specific markers, and the dysregulation of these genes may be related to retinal disease [26]. Furthermore, *RBP3* is a glycoprotein expressed in the interphotoreceptor matrix of the retina [27]; it is also associated with retinitis pigmentosa [28]. Interestingly, the clinical signs of retinopathy in OXYS rats appear by approximately 3 months of age. The phototransduction genes such as *GNAT2* and *OPN1SW* and eye development genes such as *GNAT2* and *RBP3* are unexpectedly upregulated in OXYS rats at 3 months of age [29]. Hence, the dysregulated *GNAT2* and *RBP3* associated with *OPN1SW* might jointly function in the ONH and contribute to the development of POAG.

Notably, the TF-DEG network demonstrated that downregulated *NEUROD1* was significantly linked with 36 TFs, and this gene was identified in the DEG interaction network. Moreover, *NEUROD1* promotes the formation of early retinal ganglion cells [30], and retinal ganglion cell counts are associated with early visual field defects of glaucoma [31]. Thus, *NEUROD1* might be a key marker gene in the ONH of POAG patients. Early studies on cultured retinal cells have shown that loss of *NEUROD1* causes delayed amacrine differentiation, increased bipolar cell population, death of a subset of rod photoreceptors, and increased gliogenesis [32]. Furthermore, knockout of *NEUROD1* in mice highlighted a role of this gene in long-term maintenance and survival of photoreceptors and photoreceptor differentiation [33]. More recently, targeted gene deletion studies showed that *NEUROD1* is required for the survival of photoreceptors, but not pinealocytes, indicating a specific role for this gene in photoreceptors [34]. Photoreceptors are affected by chronically elevated intraocular pressure and are associated with glaucoma [35]. Optical coherence tomography studies showed that eye damage in glaucoma patients related to structural changes in the photoreceptor layer [36]. This highlighted a crucial role of *NEUROD1* in POAG.

In conclusion, we identified 31 significant DEGs between normal ONH and the ONH of patients with POAG based on gene expression profiling. Further, network and TF prediction analyses revealed genes with abnormal expression, including *GNAT2*, *RBP3*, and *NEUROD1*, which might have important implications in POAG. These genes, especially *NEUROD1*, are involved in different eye diseases. At the genetic level, the presence of abnormally expressed genes further confirmed the hypothesis that the ONH is closely related to the occurrence of POAG. Moreover, our analyses may provide a basis for developing novel therapies for POAG. However, more in-depth experimental studies (such as real-time quantitative polymerase chain reaction) are needed to verify our findings.

## Disclosure

Xinrong Wang and Ke Gong are co-first authors.

## Figures and Tables

**Table 1:** The 31 significant DEGs between POAG ONH and normal ONH samples.

Gene	logFC	*P* value
*HIST1H2BE*	1.085616	0.018774
*ST13P4*	0.873623	0.00046
*HIST1H2AJ*	0.778174	0.025284
*AGXT2L1*	0.774778	0.028185
*PTGS2*	0.71795	0.01722
*ASPN*	0.713362	0.030858
*PSME2*	0.65883	0.030883
*ULBP1*	0.648397	0.031441
*DPPA3*	0.636051	0.009133
*FLJ37543*	0.594389	0.034072
*ITGBL1*	0.585321	0.027386
*FABP3*	−0.60266	0.014561
*FSTL5*	−0.60642	0.00971
*IMPG2*	−0.61099	0.027252
*DNM1P41*	−0.61537	0.005843
*RBP3*	−0.62018	0.017575
*RARRES1*	−0.63046	0.02175
*OR2M7*	−0.65331	0.02169
*OR2V2*	−0.68567	0.000135
*APIP*	−0.71479	0.03663
*EYS*	−0.72694	0.025346
*NEUROD1*	−0.72925	0.043711
*OR13C9*	−0.74864	0.006745
*GNAT2*	−0.74885	0.011052
*IMPG1*	−0.77855	0.025706
*UBQLN4*	−0.79455	0.017207
*CLUL1*	−0.81503	0.037454
*SUMO1P3*	−0.82912	0.015582
*OR4N4*	−0.83176	0.013802
*GUCA1C*	−0.87337	0.035767
*OPN1LW*	−1.09261	0.009174

DEGs, differentially expressed genes; POAG, primary open-angle glaucoma; ONH, optic nerve head; FC, fold change.

**Table 2:** GO and KEGG pathway enrichment analysis of DEGs.

Category	Ontology/pathway	ID	Term	Gene count	*P* value
POAG-UP	BP	GO:0006334	Nucleosome assembly	2	0.030668023
	BP	GO:0031497	Chromatin assembly	2	0.031749223
	BP	GO:0065004	Protein-DNA complex assembly	2	0.033189322
	BP	GO:0034728	Nucleosome organization	2	0.033908729
	CC	GO:0000786	Nucleosome	2	0.038767339

POAG-DOWN	BP	GO:0007600	Sensory perception	11	3.23E-09
	BP	GO:0050890	Cognition	11	9.87E-09
	BP	GO:0007601	Visual perception	7	1.08E-07
	BP	GO:0050953	Sensory perception of light stimulus	7	1.08E-07
	BP	GO:0050877	Neurological system process	11	1.53E-07
	REACTOME_PATHWAY	REACT_14797	Signaling by GPCR	5	0.005027711
	KEGG_PATHWAY	hsa04740	Olfactory transduction	5	8.87E-04

GO, Gene Ontology; KEGG, Kyoto Encyclopedia of Genes and Genomes; DEGs, differentially expressed genes; BP, biological process; CC, cellular component.
